# Exposure to Adverse Social Behavior in the Workplace and Sickness Presenteeism among Korean Workers: The Mediating Effects of Musculoskeletal Disorders

**DOI:** 10.3390/ijerph15102198

**Published:** 2018-10-09

**Authors:** Sookja Choi, Yunjeong Yi, Jiyun Kim

**Affiliations:** 1 Red Cross College of Nursing, Chung-Ang University, Seoul 06974, Korea; sjchoi2u@cau.ac.kr; 2 Department of Nursing, Kyung-In Women’s University, Incheon 21041, Korea; yinyis@kiwu.ac.kr; 3 School of Nursing, Gachon University, Incheon 21936, Korea

**Keywords:** workplace violence, mediation, musculoskeletal diseases, presenteeism

## Abstract

Adverse social behavior (ASB) by colleagues or superiors in the workplace is considered highly stressful for workers in South Korea. The authors investigate the mechanism by which ASB reduces productivity (measured in terms of sickness presenteeism (SP)), by examining the potential mediating role of musculoskeletal disorders (MSDs). All data are derived from the fourth Korean Working Conditions Survey, which investigated a representative sample of the working population. The authors analyze their general characteristics (age, gender, income, and education), work-related factors (job type, occupational ergonomic risk, job resource, employment contract, work schedule, working hour, and job demand), and health-related factors (self-rated health and MSDs). The authors use a two-step regression analysis to estimate the direct effect of ASB on SP and the indirect effect of SP via MSDs. The authors find that MSDs mediate 16.7% of the total effect of ASB on SP. When employment type and job conditions are considered, the role of the mediating variable in the group with a permanent contract, no shift or night work, and high working time is greater than the counterpart of each variable. Various strategies are needed to address MSDs according to the working environment, which might help limit the negative impact of ASB on SP.

## 1. Introduction

Adverse social behavior (ASB) is defined as all acts of physical and verbal violence and intimidation at work [1]. This term includes the acts of bullying/harassment, and violence [2]. There are many definitions of bullying, harassment, and violence in workplace. Workplace bullying is defined as a form of long-term interpersonal aggression that can be both inhuman and persistently crafty [3]. Bullies are in superior positions and psychological bullying is more applicable to the workplace than physical bullying, usually with repeated acts [4]. Harassment is defined as interpersonal behavior aimed at intentionally harming another employee in the workplace [5]. This is somewhat different from bullying in that the harassment is specific to the victim’s sex, race, age, religion, disability, etc. [4]. Workplace violence is hard to define because this concept is very extensive and diverse according to the perspectives regarding work and violence, the culture of the workplace, working characteristics, working policies in each country, etcetera. The definition of workplace violence was defined in International Labour Office as “any action, incident or behaviour that departs from reasonable conduct in which a person is assaulted, threatened, harmed, injured in the course of, or as a direct result of, his or her work” [6]. Workplace violence includes bullying, rape, intimidation, rude gestures, etc. [7]. Therefore, bullying, harassment, and violence are not mutually exclusive alternatives. When the whole behavior, such as bullying/harassment, and violence are considered, there can be many types; physical, psychological and/or sexual; one-off incidents or more systematic patterns of behavior; amongst colleagues, between superiors and subordinates or by third parties such as clients, customers, patients, pupils, etc. [8]. They also can range from minor cases of disrespect to more serious acts, including criminal offences, which require the intervention of public authorities [8]. This can negatively affect the worker and workplace environment, so raising awareness and reducing the likelihood of these occurrences are very important [8]. 

The European Foundation for the Improvement of Living and Working Conditions (Eurofound) created an index of ASB that measures experiences of bullying, violence, and sexual harassment in the past year and/or verbal abuse, humiliating behavior, and unwanted sexual attention in the past month [1]. This indicator is a component of the social environment index, which is a sub-element for measuring job quality [9]. The results of a national survey revealed that the prevalence of ASB was 5.8% among Koreans [10], which is much lower than the prevalence among European workers, at 14.9% (based on the same questionnaire) [11]. The prevalence of specific aspects of ASB, however, appears to vary substantially depending on the study. A sample of manufacturing employees, 3.5%, reported being victims of bullying [12], which is much higher than that found in another study at 0.3% [10], for instance. 

Exposure to ASB in the workplace has been found to lead to numerous adverse health outcomes, such as sleep problems [13] and depression [14], as well as various negative job outcomes, such as greater turnover intention, decreased job satisfaction [15], and decreased productivity [16,17]. The authors thought it particularly important to focus on physical conditions, such as musculoskeletal disorders (MSDs), having a significant impact on the economy: specifically, MSDs account for 40–50% of the costs of all work-related health issues in European countries [18]. MSDs are also the most prevalent occupational disease in South Korea. MSDs accounted for 61.38% of all occupational diseases in South Korea in 2016 [19]. It is, therefore, necessary to investigate whether MSDs act as a mediator in the relationship between ASB and SP. 

To investigate why ASB is related to MSDs, an Allostatic Load model was used in this study, which explains how stress results in both adaptive and maladaptive effects [20]. Using this model, ASB can be a work stress and explained as “the process by which workplace psychological experiences and demands (stressors) produce both short-term (strains) and long-term changes in mental and physical health” [21]. When the workers experience ASB the first time, they adapt in the short-run with the mechanism of “allostasis”, during which hormones act as stress protectors of the body and promote adaptation [20]. ASB can lead to negative physical and psychosocial results, however, even if it is adaptive in the short-run. The Allostatic Load model explains this with a sequential process from acute stress-related responses of adaptation, such as secretion of cortisol and epinephrine, to longer-term processes of symptom and disease occurrence [21]. Among the physical and psychosocial outcomes in this model, MSDs such as backache were studied as an associated result with various work stressors [22].

Generally, if employees are sick due to unfair treatment at the workplace or exposure to harassment, they will not go to work. There are studies representing the status of going to work despite having exceedingly poor health, however, which is called sickness presenteeism (SP). It can lead to numerous adverse health outcomes as well as a loss of productivity [9]—naturally, if employees are in a poor condition, their working capacity will be suboptimal. Overall, 21.7% of workers in South Korea have reported working when they were sick [23]. SP in South Korea is relatively lower than that in Europe, where the prevalence was 39% (using the same questionnaire) [9]. Other surveys have not found as low levels of SP in South Korea, however. During a study on Korean railroad workers, the prevalence of SP was 52% [24]. Found in another study, the prevalence of SP was compared between people with and people without depressive symptoms. The results showed that the prevalence of SP was 43.7% among participants with depressive symptoms and 36.3% among those without [25]. The median loss of productivity costs of SP due to depression was estimated to be 1175 USD per worker [26].

Conway et al. examined bullying and workers’ coping behaviors with SP [17] in terms of a conservation of resources theory [22]. Workers who experience ASB are in a resource-loss situation and continue going to the workplace, even when they feel sick, to prevent potential loss or theft of resources. Furthermore, they found that health status is a partial mediator of the relationship between an ASB such as workplace bullying and SP [17]. The job demands-resources (JD-R) model also explained the process of ASB and presenteeism. Job demands are “physical, social, or organizational aspects of the job that require sustained physical or mental effort and are, therefore, associated with certain physiological and psychological costs (such as exhaustion) [27].” Found in JD-R, job demands are primarily and positively related to exhaustion, whereas job resources are primarily and negatively related to disengagement from work [27]. Job demand can change an employee’s health status to being impaired [28] and also cause more presenteeism [29]. McGregor et al. found that psychosocial job demands, including workplace bullying, were indirectly related to presenteeism [30]. The authors want to test a hypothesis concerning the relationship between ASB and SP, with a mediating effect of MSDs.

**H1**: *MSDs mediate the relationship between the ASB and Presenteeism.*

There are employees who are more likely to feel threatened by their social environment and tend to apply maladaptive coping strategies, like going to work sick. These phenomena can be also explained by the JD-R model [27]. Using this model, job demands which lead to exhaustion, can be represented by working hours and working schedule. Resource can be regarded as employment-type, whether a permanent contract or not. During this study, the authors will consider various work-related factors for measuring the social environment among workers, ensuring to account for these workplace characteristics when examining the mediating effect of MSDs on the association between ASB and SP. This leads to the following hypothesis:

**H2**: *The positive relationship between MSDs and SP is moderated by employment (employment contract) or job conditions (work schedule, working hour, and job demand).*

**Figure 1:**
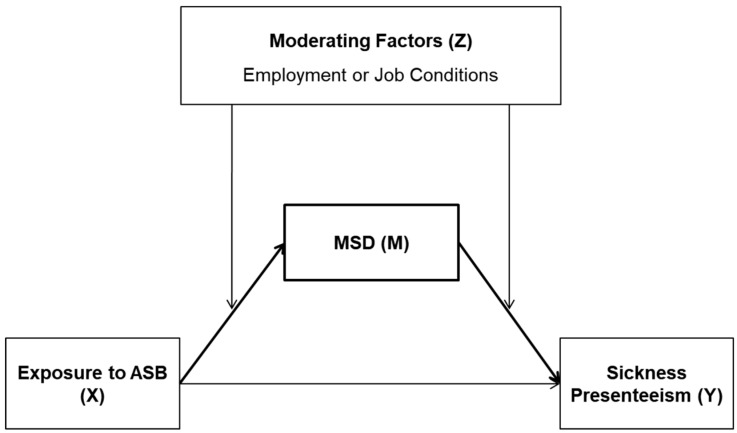
Hypothesized model for ASB, SP, MSDs, and Moderators.

During this study, the authors investigate the positive relationship between ASB and SP, as well as the direct and indirect effects of ASB on SP (Figure 1). Furthermore, the authors examine how the mediating effect of MSDs differs by work characteristics. 

## 2. Methods

### 2.1. Data

The authors used data from the fourth Korean Working Conditions Survey (KWCS), which was conducted by the Korean Occupational Safety and Health Agency (KOSHA) in 2014 on a representative sample of the working population aged ≥15 years. It used a multistage, stratified random sampling method. The authors used weighted data to reflect this sampling method and the response rate and, thereby, obtain nationally representative estimates. Altogether, 50,032 workers were surveyed through face-to-face interviews. The current study investigated the general characteristics, work-related characteristics, and health-related characteristics of workers to examine the mediating effect of MSDs on the relationship between ASB and SP. Original dataset had 50,032 participants which included various types of workers (e.g., employer, self-employed, unpaid family workers, and so on), but the analysis was limited to (30,751) waged earners. After excluding the participants (*n* = 7522) with missing values for all the variables used in this study, we included 23,229 waged workers in the final data analysis. This study was exempted from the need to obtain approval from an institutional review board (IRB no. 1041078-201803-HRSB-047-01) because all data were available to the public. 

### 2.2. Measurements

The general characteristics in this study were age, gender, monthly income, and education level. Participants were divided into four groups for both age and monthly income. Education level was categorized into three groups.

The work-related factors included job type, occupational ergonomic risk, job resource, employment contract, work schedule, working hour, and job demand. Job type was classified into ten categories according to the Korean Standard Classification of Occupation [31]. Occupational ergonomic risk was analyzed in terms of the frequency of painful or tiring positions; carrying heavy loads; lifting or moving people; standing; exposure to vibrations; and repetitive hand or arm movements. Specifically, the authors examined the proportion of the overall work time occupied by these activities using a seven-point scale that was later dichotomized as “no” (answers of “never” and “almost never”) and “yes” (answers of “around 1/4 of the time”, “around half of the time”, “around the 3/4 of the time”, “almost all the time”, and “all of the time”). 

Job resource consist of work autonomy (three items), coworker support (one item), and supervisor support (one item). Work autonomy was a three-item scale from Bakker et al. and included “You have a say in the choice of your working partners”, “You can take a break when you wish”, and “You are able to apply your own ideas in your work” [32]. Coworker support was measured with one item modified from Bakker et al. which included the question “Your colleagues help and support you” [32]. Supervisor support was one item modified from Demerouti et al. with the inclusion of “Your manager helps and supports you” [27]. The response options were: “never”, “rarely”, “sometimes”, “most of the time”, and “always”, with each response graded as 1, 1.25, 1.50, 1.75, 2.0 respectively. The authors utilized the summation of these three dimensions (Cronbach’s alpha = 0.74).

Employment contract was categorized into two categories: permanent (≥1 year, no fixed term) and short term (<1 year, fixed term). Work schedule was divided into two categories (shift/night work and daytime work), while working hours (the total working hours per week) were classified into 4 categories. Job demands utilized three items from Caplan et al. including “Does your job involve working at very high speed?”, “Does your job involve working to tight deadlines?” and “How often do you have to interrupt a task you are doing to take on an unforeseen task?” [33]. This study used a response scale for measuring a high speed and tight deadline using a seven point scale range from 1 to 2: 1 (never), 1.17 (almost never), 1.33 (around 1/4 of the time), 1.50 (around half of the time), 1.67 (around 3/4 of the time), 1.83 (almost all the time), 2 (all of the time) and also utilized measuring for an interruption of a task using 1 for “never”, 1.33 for “occasionally”, 1.67 for “fairly often”, and 2 for “very often” (Cronbach’s alpha = 0.67).

The health-related factors assessed were self-rated health and MSDs. Self-rated health was measured with a single item: “How would you rate your general state of health?” This was rated using five response options, which were later grouped into “very good/good” and “fair/bad/very bad”. MSDs were assessed using three items related to three different parts of the body: backache, upper limb complaints (muscular pain in the shoulders, neck, and/or upper limbs [arms, elbows, wrists, and hands]), and lower limb complaints (muscular pain in the lower limbs [hips, legs, knees, and feet]). The items were prefaced with a single question: “Over the last 12 months, have you had any of the following health problems?” Possible answers were “yes” or “no”. The authors coded 0 for “no” and 1 for “yes” and then MSDs were calculated by summation of three items.

ASB was assessed using six items, each answered with “yes” or “no”. The employees were asked whether they had experienced: (1) verbal abuse, (2) unwanted sexual attention, or (3) threats and behavior intended to humiliate them over the last month, as well as (4) physical violence, (5) bullying/harassment, or (6) sexual harassment over the past 12 months. ASB also was calculated by the same method of computing MSDs. When the worker answered the ASB questionnaire, the number of experiences of ASB were between 0–6.

Finally, SP was measured by asking respondents to state how frequently these behaviors happened in a given period: “How many times during the past 12 months have you gone to work despite feeling that you really should have taken sick leave due to your state of health?”. This item was modified from a previous study [34]. The current authors used this self-reporting since its measure is arguably the most commonly used in studies on SP [35]. During this study, the mean presenteeism was 1.19 with a standard deviation of 6.61. The authors used square root transformation to account for truncated and positively skewed data. 

### 2.3. Statistical Analysis

The distribution of ASB was classified according to the general and work-related characteristics of the study sample. To examine the difference in experiences of ASB by general characteristics, the authors used the χ^2^ test. The correlations among ASB, MSDs, SP and moderator variables were examined using Spearman’s correlation.

To investigate MSDs as a possible mediator between ASB and SP (Hypothesis 1), the authors followed the steps recommended by Preacher and Hayes and used bootstrapping to obtain estimates of the indirect (mediating) effects [36]. This approach has been shown to be superior to other forms of detecting mediation [36]. According to this analytical approach, the current study utilized 1000 bootstrapping samples with 95% bias-corrected confidence intervals to test the significance of these effects (direct, indirect, and total effect). Bootstrapping is a statistical method of estimating the sampling distribution of an estimator by sampling with replacements from the original sample—the main purpose of this is to derive robust estimates of the standard errors and confidence intervals of a population parameter (mean, median, proportion, odds ratio, correlation coefficient, or regression coefficient). Bootstrapping has been shown to be a good method for testing significance in mediation models as it makes no a priori assumptions regarding the normality of the distribution of the variables tested [36]. This assumption of normality is often violated when examining indirect effects (the product of the coefficients for path a and path b) in mediation models [37]. To test mediation using this approach, two equations were used:

MSD = α_0_ + α_1_ASB + α_2_ covariates + ν (1)

SP = β_0_ + β_1_ASB + β_2_MSD + β_3_covariates + ε (2)

Hypothesis 2 indicated the employment condition (employment contract) or job conditions (working hours, work schedule, and job demand) would influence the mediating effect of MSDs on the relationship between experiencing the adverse social behavior and sickness presenteeism. To test for moderated-mediation, the authors utilized the approach suggested by Edwards and Lambert (2007) [38], which allowed testing of the stage 1 moderation model and stage 2 moderation, total effects model. This approach also allowed for the examination of the indirect and total effects for the models using 1000 bootstrapping samples and 95% bias-corrected confidence intervals. To analyze moderated-mediation using this approach, the authors calculated the following equations:

MSD = α_0_ + α_1_ASB + α_2_Z + α_3_ASB × Z + α_4_covariates + ν (3)

SP = β_0_ + β_1_ASB + β_2_MSD + β_3_Z + β_4_ASB × Z + β_5_MSD × Z + β_6_covariates + ε (4)

Z is employment or job conditions in these equations. The results from Equation (3) are then plugged into Equation (4) to provide the intercept and slope of this moderated-mediation model. 

Prior to analysis, all continuous measures were mean centered, whereas all dichotomized moderator variables were coded 0 and 1. When Z was a continuous variable (working hour, job demand), the representative scores in the distribution of Z, such as one standard deviation above and below its mean were utilized [39]. When Z was a categorical variable (employment contract, work schedule), the score used to code Z was used to obtain the simple effects for each category. Simple effects represented by single paths were tested with procedures for simple slope [38].

All relationships were adjusted in the analysis by considering some relevant confounders, including employees’ general characteristics and work-related factors. 

A *p* < 0.05 was considered statistically significant (two-tailed test). The statistical analyses were performed using SPSS 23 (SPSS, Inc., Chicago, IL, USA).

## 3. Results

The distribution of the sample characteristics and the prevalence of ASB are shown in Table 1. The sample comprised 12,081 male workers and 11,264 female workers, and about 70% of all subjects were aged 30–64 years. Half of the respondents had graduated from college/university and beyond, and 63% earned a monthly income of 1–2.99 million KRW. The four most dominant job types were clerical (26.9%), unskilled worker (15.0%), service (13.5%), and sales (12.2%). A quarter of the respondents were short-term or fixed-term workers. Most of the participants worked for ≤52 h per week (82.3%) and daytime work (84.4%). The majority of workers reported having at least one occupational ergonomic risk factor (84.3%). Three-quarters of the subjects self-reported their general health was good. 

The overall prevalence of ASB and SP were 7.0% and 22.2%, respectively. More specifically, for ASB, 1642 respondents (7.0%) reported “yes” to at least one of the six items, and 21,600 respondents (93.0%) did not. Regarding MSDs, they were generally highly prevalent among respondents (40.0%), whereas that for SP was 22.2%.

Information on the descriptive statistics and correlation analyses are shown in Table 2. The authors examined the correlations between SP, ASB, the potential mediator (MSD) and moderator variables (employment contract, work schedule, working hour, and job demand). It was noted that ASB (r = 0.14, *p* < 0.001) and MSD (r = 0.27, *p* < 0.001) were positively related to presenteeism. All moderator variables were significantly related with ASB, MSDs, and SP. 

The simple mediation results of the hypothesized model are provided in Table 3. The authors found that MSDs mediated the relationship between ASB and SP. The authors found a significant total (point estimate = 0.24, *p* < 0.001) and indirect effect (point estimate = 0.04, *p* < 0.001). Additionally, we add the full regression tables for predicting MSD (Model 1) and SP (Model 2) in the Appendix A (Table A1).

Table 4 shows the total effect was dominated by the direct effect on the moderated mediation effect of MSDs on the ASB and SP relationship. More specifically, the role of the mediating variable in the group with a permanent contract, no shift or night work, and high working time was greater than the counterpart of each variable.

**Figure 2:**
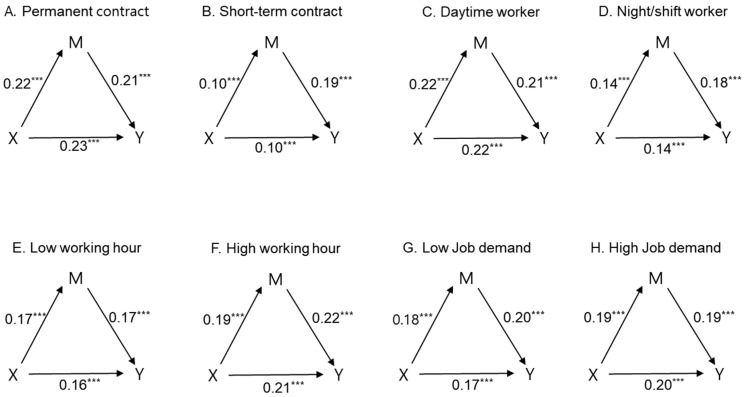
Mediated models showing simple effect for each moderator variable. Mediated models showing simple effects for each moderator variable. Regarding each model, X, M, and Y represent ASB, MSDs, and SP, respectively. Coefficients in boldface were significantly different at *p* < 0.001 across the level of the moderator variable (Employment contract for Panels **A** and **B**, Work schedule for Panels **C** and **D**, Working hour for Panels **E** and **F**, Job demand for Panels **G** and **H**).

Figure 2, Panels A and B show an employment contract moderated the paths from ASB to MSDs and MSDs to SP, both of which were larger for a permanent contractor than for a short-term contractor. This same tendency appeared in work schedule (daytime worker > night or shift worker) and working hours (longer working hour worker > shorter working hour worker) except regarding job demand. 

## 4. Discussion

The authors investigated the mediating effect of MSDs on the relationship between ASB and SP among Korean workers in this study. The results showed that all the MSDs mediated the relationship between ASB and SP. Specifically, the size (percentage of total effect mediated) of the mediating effect of MSDs on the relationship between ASB and SP was 16.7% and this result provided support for Hypothesis 1. Many previous studies investigated the relationship of MSDs with SP [40,41] and productivity loss [18]. The current findings, in general, coincide with these past studies, which showed that employees who have experienced ASB tend to have a poorer health status [42] and a higher probability of SP [43]. These findings also expand on those of previous studies, which found a significant relationship between ASB (physical violence, sexual harassment, and harassment) and SP [23]. SP might interrupt recovery from MSDs and contribute to the aggravation induced by ASB [17]. 

This, perhaps, is because under such conditions, even workers suffering from an MSD attend work despite their poor physical conditions. While the occurrence of MSDs largely depends on the nature of the work, such as mechanical pressure concentration, type of occupation, and type of work, abuse or violence can also lead to MSDs by causing tension or strain on workers [18]. Therefore, it is necessary to scrutinize the occurrence of MSDs among workers in favorable circumstances as well as in poor occupational environments.

The authors also analyzed how the mediating effect of MSDs on the relationship between ASB and SP differed according to employment conditions or job conditions. The results showed that an employment contract moderated the paths from ASB to MSDs and MSDs to SP, both of which were larger for a permanent contractor than for a short term contractor. SP is known to be related to various workplace characteristics such as working hours, work schedule, lack of reward [44], and job strain [23], in South Korea. Additionally, ASB has been found to relate to employment contract, work schedule, and working hour [10]. It is meaningful that the total effect on a permanent contractor who is a victim of ASB has a higher SP than on a short-term contractor. It is hard to dismiss a permanent contractor in Korea and companies pay permanent contractors relatively higher wages than part-time contractors [45]. Moreover, there is little opportunity for part-time contractors to attain permanent employment [45]. Therefore, permanent contractors seem to try to avoid loss of potential resources while suffering ASB to maintain their work life with SP. Although SP can be seen to be a sign of high commitment, it ultimately leads to low productivity. These results indicate that the working characteristics should be considered when confirming the relationship between the ASB and the SP.

### 4.1. Research Limitations and Recommendations for Future Research

This study has some limitations. First, this investigation of the mediating effect of MSDs on the relationship between ASB and SB was cross-sectional and, therefore, causal relationships cannot be inferred from the results. Second, while instruments are being developed to measure SP and the resulting productivity loss [46], the authors could only use an earlier questionnaire to measure SP due to a reliance on secondary data. Moreover, the authors could not add other variables to adjust the analysis. Future research should investigate SP while considering productivity loss. Third, the authors failed to measure MSDs objectively (again, due to using secondary data). Last, the authors did not consider common method variance that is a potential problem in organizational and behavioral research. These method biases arise from having a common rater, a common measurement context, a common item context or from the characteristics of the items themselves [47]. During any given study, it is possible for several of these factors to be operative through the design of the study’s procedures and/or statistical control, but in this study, the authors could not consider these biases which may inflate relationships between variables. Despite these limitations, the authors were able to delineate a possible mechanism (MSD) by which ASB relates to SP among Korean workers. A strength of this study was its control of health-related factors such as self-rated health and job strain. While the strength of the relationship between ASB and SP was somewhat smaller than the relationship between MSDs and SP, the former was nevertheless significant. This reinforces the relevance of ASB exposure to going to work while ill.

### 4.2. Research Contributions

This study focused on the association of workplace ASB as a physical risk factor for SP. When workers attend their workplace in a suboptimal condition—such as when they present physical problems (MSD for example)—occupational nurses or supervisors of the workers’ departments should assess their general health conditions, including whether they are victims of ASB. 

## 5. Conclusions

This study showed that workers exposed to ASB reveal an increase in SP if there is an MSD. Some studies have tested possible interventions for MSDs to help prevent productivity loss. The use of ergonomic services, or ergonomic services plus a trackball or forearm support, did not lead to significant improvement in productivity loss compared to a control group [48]. Notably, one study found that if an organization has a strong culture of presenteeism, MSDs are likely to have a stronger relationship with SP [49]. Thus, the prevention of productivity loss due to MSDs might involve not only ergonomic mediation but also improving the workplace atmosphere, particularly the incidence of ASB.

## Figures and Tables

**Table 1:** Distribution of study population and prevalence of ASB, SP, and MSDs by key covariates (*N* = 23,229).

Variable	Category	Distribution		ASB			SP			MSDs		
		*n*	%	%	χ^2^	*p*	%	χ^2^	*p*	%	χ^2^	*p*
				7.0			22.3			40.3		
(years)	≤29	5504	23.6	8.3	21.98	<0.001	18.0	83.84	<0.001	27.3	1028.18	<0.001
	30–49	9816	42.1	6.7			23.8			37.0		
	50–64	6221	26.7	6.7			23.9			49.5		
	Over 65	1775	7.6	5.6			21.0			62.9		
Gender	Male	12,081	52.0	6.4	17.58	<0.001	20.1	62.88	<0.001	35.5	212.03	<0.001
	Female	11,264	48.0	7.7			24.4			44.8		
Income (monthly, million Korean won *)	<1	2985	12.8	6.8	70.31	<0.001	16.8	65.42	<0.001	49.3	310.71	<0.001
	1–1.99	8307	35.6	8.8			24.0			43.9		
	2–2.99	6427	27.5	6.3			22.6			37.1		
	≥3	5630	24.1	5.4			21.9			32.6		
Education	Below middle school	2783	11.9	7.2	43.89	<0.001	24.2	11.91	0.032	65.4	1150.50	<0.001
	High school	8856	37.9	8.4			22.6			43.7		
	College/university and beyond	11,711	50.2	6.0			21.4			31.2		
Job type	Senior manager	460	1.9	4.1	242.60	<0.001	17.8	69.29	<0.001	25.0	1298.35	<0.001
	Professional	1972	8.4	5.4			25.2			33.3		
	Technicians	1232	5.3	5.1			23.0			32.7		
	Clerks	6251	26.8	4.8			19.5			27.5		
	Service	3164	13.5	11.6			23.7			48.9		
	Sales	2868	12.3	10.6			20.3			33.9		
	Agriculture or fisheries	90	0.4	3.3			24.4			65.6		
	Craft worker	2316	9.9	5.0			24.6			48.5		
	Operators	1488	6.4	7.8			25.1			46.2		
	Unskilled workers	3508	15.0	7.1			23.0			58.7		
Occupational ergonomic risk	Yes	19,683	84.3	7.5	49.30	<0.001	23.6	143.81	<0.001	43.9	816.13	<0.001
	No	3666	15.7	4.3			14.6			18.7		
Job resource	Low	13,567	58.1	8.1	51.92	<0.001	23.2	14.97	0.001	44.5	266.02	<0.001
	High	9782	41.9	5.6			21.0			33.7		
Employment contract	permanent contract	17,508	75.0	6.5	33.02	<0.001	23.1	29.37	<0.001	37.2	224.95	<0.001
	short-term contract	5841	25.0	8.7			19.6			48.3		
Work schedule	Shift/night work	3653	15.6	13.3	256.01	<0.001	25.7	31.16	<0.001	43.3	20.19	<0.001
	Daytime work	19,696	84.4	5.9			21.7			39.7		
Working hour (weekly)	≤40	12,700	54.4	5.7	120.84	<0.001	19.7	135.62	<0.001	36.8	169.31	<0.001
	41–52	6522	27.9	7.4			23.7			41.5		
	53–60	2910	12.5	10.2			28.8			47.6		
	≥61	1217	5.2	11.6			26.0			48.2		
Job demand	Low	14,427	61.8	5.3	184.06	<0.001	19.2	193.01	<0.001	36.2	236.46	<0.001
	High	8922	38.2	9.9			27.0			46.2		
Self-rated health	Good	17,189	73.6	5.9	137.35	<0.001	18.3	587.74	<0.001	31.0	2127.38	<0.001
	Bad	6160	26.4	10.3			33.4			65.0		
ASB	Yes	1642	7.0									
	No	21,707	93.0									
MSDs	Yes	9337	40.0	10.4	274.97	<0.001	34.7	1401.11	<0.001			
	no	14,012	60.0	4.8			13.9					
SP	yes	5185	22.2	13.1	379.54	<0.001				62.8	1399.43	<0.001
	no	18,164	77.8	5.3						33.8		

NOTE: ASB = adverse social behavior; MSDs = musculoskeletal disorders; SP = sickness presenteeism; * 1 million Korean won ≈ 891.95 US.

**Table 2:** Means, Standard deviation, and Correlations matrix of the predictor, mediator, moderators, and outcome.

Variables	M	SD	Range	1	2	3	4	5	6	7
1.ASB	0.11	0.50	0–6							
2.SP	1.19	6.62	0–362	0.14 ***						
3.MSDs	0.70	0.98	0–3	0.12 ***	0.27 ***					
4.Employment contract	0.25	0.43	0–1	0.04 ***	–0.03 ***	0.12 ***				
5.Work schedule	0.16	0.36	0–1	0.11 ***	0.04 ***	0.03 ***	–0.01			
6.Working hour	43.64	12.58	2–144	0.06 ***	0.08 ***	0.05 ***	–0.22 ***	0.15 ***		
7.Job demand	3.74	0.58	3–6	0.10 ***	0.09 ***	0.12 ***	–0.01	0.10 ***	0.14 ***	

Note: ASB = adverse social behavior; SP = sickness presenteeism; MSDs = musculoskeletal disorders. The number in parentheses is the coefficient alpha. * *p* < 0.05, ** *p* < 0.01, *** *p* < 0.001.

**Table 3:** Total, direct, and indirect effect of adverse social behavior on sickness presenteeism (Mediation model).

Mediator	Direct Effect (CI)	Indirect Effect (CI)	Total Effect (CI)	% of Total Effect Mediated
MSD	0.20 *** (0.15, 0.24)	0.04 *** (0.03, 0.05)	0.24 *** (0.19, 0.28)	16.7

Note: MSD = musculoskeletal disorders. Adjusted for age, gender, income, education, job type, occupational ergonomic risk, job resource, employment contract, work schedule, working hour, job demand, and self-rated health. Bootstrap confidence intervals were constructed using 1000 resamples and 95% bias-corrected confidence intervals. * *p* < 0.05, ** *p* < 0.01, *** *p* < 0.001.

**Table 4:** Results of the moderated mediation analysis.

Moderator Variable		Stage		Effect		
		First	Second	Direct	Indirect	Total
Employment contract	permanent contract	0.22 ***	0.21 ***	0.23 ***	0.05 ***	0.28 ***
	short-term contract	0.10 ***	0.19 ***	0.10 ***	0.02 ***	0.12 ***
	DIFFERENCE	0.13 ***	0.02	0.13 ***	0.03 ***	0.16 ***
Work schedule	daytime	0.22 ***	0.21 ***	0.22 ***	0.04 ***	0.27 ***
	night/shift	0.14 ***	0.18 ***	0.14 ***	0.02 ***	0.16 ***
	DIFFERENCE	0.08 **	0.03	0.09 **	0.02 **	0.10 ***
Working hour	−1 SD	0.17 ***	0.17 ***	0.16 ***	0.03 ***	0.19 ***
	+1 SD	0.19 ***	0.22 ***	0.21 ***	0.04 ***	0.25 ***
	DIFFERENCE	0.03	0.05 ***	0.05 *	0.01 **	0.06 **
Job demand	−1 SD	0.18 ***	0.20 ***	0.17 ***	0.04 ***	0.21 ***
	+1 SD	0.19 ***	0.19 ***	0.20 ***	0.04 ***	0.23 ***
	DIFFERENCE	0.01	0.01	0.03	0.00	0.03

Note: *N* = 23,229. Adjusted for age, gender, income, education, job type, occupational ergonomic risk, job resource, and self-rated health. Bootstrap confidence intervals were constructed using 1000 resamples and 95% bias-corrected confidence intervals. Regarding rows labeled, all moderator variables are simple effects computed from Equations (3) and (4). First stage indirect effect = α_1_ + α_3_ Z. Second stage indirect effect = β_2_ + β_5_Z. Direct effect = β_1_ + β_5_Z. Indirect effect = (α_1_ + β_4_Z) (β_2_ + β_5_Z) Total effect = Direct + Indirect * *p* < 0.05, ** *p* < 0.01, *** *p* < 0.001.

## Appendix A

**Table A1:** Simple mediation Results.

Variable		Model 1	Model 2
Predictor		MSDs B(SE)	Presenteeism B(SE)
*Control variable*			
Age		0.01 (0.00) ***	0.00 (0.00)
Gender (ref = male)		0.20 (0.01) ***	0.08 (0.01) ***
Income (ref: <1 million Korean won)			
	1–1.99	0.04 (0.02)	0.09 (0.03) **
	2–2.99	0.08 (0.03) **	0.09 (0.03) **
	≥3	0.05 (0.03)	0.12 (0.03) ***
Education (ref = Below middle school)			
	High school	−0.16 (0.02) ***	−0.02 (0.03)
	College/university and beyond	−0.20 (0.03) ***	−0.01 (0.03)
Job type (ref = professional)			
	Senior manager	−0.05 (0.05)	−0.07 (0.05)
	Technicians	0.00 (0.03)	−0.03 (0.03)
	Clerks	−0.05 (0.02) *	−0.06 (0.03) *
	Service	0.16 (0.03) ***	−0.14 (0.03) ***
	Sales	−0.04 (0.03)	0.12 (0.03) ***
	Agriculture or fisheries	0.23 (0.10) *	0.00 (0.10)
	Craft worker	0.19 (0.03) ***	−0.07 (0.03) *
	Operators	0.14 (0.03) ***	−0.06 (0.04)
	Unskilled workers	0.19 (0.03) ***	−0.11 (0.03) **
Occupational ergonomic risk (ref = no)		0.25 (0.02) ***	0.06 (0.02) ***
Job resource		−0.03 (0.01) ***	0.01 (0.01)
Employment contract (ref = permanent)		0.00 (0.02)	−0.04 (0.02) *
Work schedule (ref = shift/night work)		−0.04 (0.02) *	0.02 (0.02)
Working hour (weekly)		0.00 (0.00) ***	0.00 (0.00) ***
Job demand		0.11 (0.01) ***	0.06 (0.01) ***
Self-rated health (ref = good)		0.55 (0.01) ***	0.26 (0.02) ***
*Independent variable*			
ASB		0.18(0.01) ***	0.19 (0.01) ***
MSDs		-	0.20(0.01) ***
R^2^		0.21 ***	0.10 ***

NOTE: ASB = adverse social behavior; MSDs = musculoskeletal disorders; SP = sickness presenteeism; Unstandardized regression coefficients are reported; Standard Errors in parentheses. * *p* < 0.05, ** *p* < 0.01, *** *p* < 0.001.
